# Examination of the Sprinting and Jumping Force-Velocity Profiles in Young Soccer Players at Different Maturational Stages

**DOI:** 10.3390/ijerph18094646

**Published:** 2021-04-27

**Authors:** Luis Miguel Fernández-Galván, Daniel Boullosa, Pedro Jiménez-Reyes, Víctor Cuadrado-Peñafiel, Arturo Casado

**Affiliations:** 1 Education Faculty, Autónoma University of Madrid, 28049 Madrid, Spain; luisdepucela@gmail.com (L.M.F.-G.); victor.cuadrado@uam.es (V.C.-P.); 2 Instituto Integrado de Saúde (INISA), Federal University of Mato Grosso do Sul, Campo Grande 79070-900, Brazil; daniel.boullosa@gmail.com; 3 College of Healthcare Sciences, James Cook University, Townsville 4811, Australia; 4 Centre for Sport Studies, Rey Juan Carlos University, 28943 Madrid, Spain; pedro.jimenezr@urjc.es; 5 Faculty of Health Sciences, Isabel I de Castilla International University, 09003 Burgos, Spain

**Keywords:** young soccer players, maturation, force-velocity profile, sprint, vertical jump

## Abstract

The aim was to determine the relationships among components of the force-velocity (F-V) profiles in jumping and sprinting, with both biological and chronological ages in 89 young soccer players belonging to categories from U10 to U18. Participants performed countermovement jumps (CMJ) and 20-m sprint tests. F-V components assessed were associated with both maturity offset and chronological age, using correlation and multiple linear regression analyses. Horizontal (i.e., maximal theoretical force [F_0_] and velocity [V_0_], maximal power [P*_max_*] and F-V slope) and vertical (i.e., [F_0_] and [P*_max_*]) F-V components displayed very large correlations (i.e., 0.79 ≤ r ≤ 0.92) with both chronological age and maturity offset. The combination of sprinting P*_max_* and training experience and jumping F_0_ and training experience explained up to 94% of the variances in maturity offset and chronological age. Furthermore, similar correlations were found between sprinting and jumping performances, and components of the F-V profiles, and both maturity offset and chronological age. Identification of vertical jump and sprint mechanical determinants may assist in strengthening those components of the F-V profile which are weaker throughout the training process. Sprinting and jumping capabilities can be indistinctly monitored with respect to their chronological age or maturity offset in young soccer players.

## 1. Introduction

The macroscopic approach to assess sprinting and jumping abilities based on the force-velocity (F-V) relationship has gained increasing attention in the sport science community [1,2]. The horizontal and vertical F-V profiles are used to measure the individual performance characteristics displayed in horizontal sprint and vertical jump tests [2,3]. The F-V relationship can be modelled from the measurement of the force and velocity performed during vertical jumps against two or more loads [4,5] and from the velocity during a maximal horizontal sprint test [2]. Recent studies have demonstrated the optimal development of jumping and sprinting performances during the training process through the use of the F-V profile in adults [1,6]. Previous studies have confirmed the link between athletes’ performance in different sports with the different mechanical variables of the F-V profiles generated from jumping and sprinting tests [7,8,9]. Several studies have already reported the F-V profile of soccer players [9,10,11,12], providing reference values of the sprint F-V profile of soccer players according to age, gender, and level of practice [7,13]. The F-V profile in sprinting and jumping is composed of different variables, such as the maximal theoretical force (F_0_), maximal theoretical velocity (V_0_), the slope of the F-V relationship (F-V slope), and maximal power (P*_max_*). In addition, the F-V profile in sprinting includes the percentage of the resultant force that is generated in the horizontal direction [2], with the decrease in the ratio of horizontal-to-resultant force (DRF) and the maximal ratio of horizontal-to-resultant force (RF peak) being typically used to assess mechanical effectiveness and sprint performance [14].

It is well known that the most relevant determining events during soccer matches are determined by high intensity actions such as jumping, changes of direction, and sprints [15]. For this reason, a typical test battery in soccer players includes different sprinting and jumping tests [16]. Specifically, for young soccer players, the measurement of these capabilities is more accurate when they are related to maturation rather than to chronological age [17]. Biological maturation, in relation to human growth, refers to the time required and the process of change until the adult maturation state is reached [18]. The physical and physiological changes that occur during the progress of biological maturation evolve at a different pace, depending on the subject [18], with the maturity offset which is defined as the time before or after peak height velocity (PHV) being one of the most useful indicators of the maturation stage [18,19]. A preliminary investigation identified the relationship between different sprinting abilities, including acceleration and maximum sprint velocity and maturity offset, but not chronological age [20]. Subsequently, some studies have found high correlations between the improved performance in sprinting and jumping in young soccer players and maturity offset [20,21,22,23,24,25], therefore highlighting sprinting capabilities as valid indicators to be used as talent-identification criteria in young soccer players. However, to date, no previous studies have either used the F-V profiles in young soccer players, or assessed the relationship between these components and both chronological age and maturity offset. Evolution of different components of the horizontal and vertical F-V profile across the maturation process in this population would assist in the development of jumping and sprinting performances, therefore providing reference values to identify the strengths and weaknesses of young soccer players with different maturational levels. In addition, this information may help to improve the validity of sprinting and jumping performance evaluations. In this way, the assessment of the evolution of the different F-V profile components would indicate the specific development state in some of the most important conditional capacities in young soccer players. Furthermore, this evaluation would provide a talent identification criterion which may prove useful for soccer teams. In this manner, optimized early identification of young talents would promote the development of any soccer club, as it brings advantages such as specialization in the skills and abilities of the sport, future economic sales, and the incorporation of players in the first team [26,27,28].

Both sprinting and jumping performances have been strongly associated with their F-V mechanical determinants in adults [5,9]. Moreover, sprinting and jumping performances have been found to change according to maturation in young soccer players [20,23,24]. Therefore, it would be reasonable to assume that the changes of the components of the F-V profiles are more related to changes in maturation than changes in chronological age. Previously, Murtagh et al. [29] reported that sprinting ability was more related to maturation changes than jumping. 

Therefore, the aims of this study were: (i) to examine the relationship between performance and F-V jump and sprint profile, with chronological age and maturity offset of young soccer players; and (ii) to compare relationships between maturity offset and chronological age for jumping and sprinting.

## 2. Methods

### 2.1. Subjects

In this study, 89 male young soccer players who train regularly in the same professional soccer academy volunteered for participation (age range: 8.04–17.93 years). Players regularly trained during at least one year prior to participation. Players trained specifically for soccer in a regular basis for 4.22 ± 2.34 years. Players and their parents agreed to participate in the current research and parental consents were signed following the tenets of the Declaration of Helsinki.

### 2.2. Design

A cross-sectional design was conducted with jumping and sprinting performances. Countermovement jumps (CMJ) without load and with two different loads (i.e., 25% and 50% of body mass), and 20-m sprints were conducted to identify the different components of the vertical and the horizontal F-V profiles, respectively (i.e., F_0_, V_0_, P*_max_*, F-V slope, F-V deficit, RF peak and DRF), according to Samozino’s method [2]. These variables were subsequently associated to both maturity offset and chronological age, with correlation and multiple linear regression analyses.

### 2.3. Methodology

Prior to data collection, anthropometric variables were recorded in the laboratory. Body mass (Tanita BF-522W, 0.1 kg precision, Japan), stature and seated height (non-commercial portable stadiometer, 0.1 cm precision) were recorded and subsequently used to calculate maturity offset through an estimation of the time before or after PHV, which also includes date of birth and the date of measurement [19]. This method approximates the amount of time (in years) until, or since, an individual’s predicted PHV indicating the maturity offset [19]. The equation used is the following:

Maturity offset = −[9.236 + 0.0002708 * Leg Length and Sitting Height interaction] − [0.001663 * Age and Leg Length interaction] + [0.007216 * Age and Sitting Height interaction] + [0.02292 * Weight by Height ratio].(1)

The distance range to PHV in order to classify soccer players by maturity status was larger than that used in previous studies using age at PHV [30], assuming the limitations of the offset equations [31,32] 

Participants were instructed to arrive for performance testing in a rested state, while avoiding strenuous exercise in the previous 48 hrs. All testing sessions were completed under thermoneutral conditions (sunny, wind speed of 1.1 ± 0.9 m/s, 23 ± 0.7 °C, and 45 ± 6.3% of relative humidity). The testing sessions were performed at 5 p.m., which is the time at which players usually train. Participants performed a standardized 15-min warm-up consisting of 5 min of jogging and 5 min of lower limb dynamic stretching followed by 5 unloaded CMJ and 3 loaded CMJs with 30 kg, and 3 progressive 30-m sprints (at 50%, 70% and 90% of the players’ self-perceived maximal velocity).

Individual F-V profiles and P*_max_* can be easily determined through the completion of a certain number of series of loaded vertical jumps [2,3,5,33]. After the warm-up, each player performed 2 CMJs (without external load, and with 2 external loads of 25% and 50% of body mass) in randomized order. Players were instructed to stand up straight with their hands on their hips for the unloaded CMJs, and on the free-weight bar (0.5 kg) for the loaded CMJs, which were performed with a knee angle of approximately ~90°. The players were instructed to jump “as high as possible.” Two valid attempts were performed with each load with 2 min of recovery between attempts, and 4–5 min between loads. Jump height was recorded using the MyJump2 app [34] installed on an iPhone 7 (Apple Inc., Cupertino, CA, USA). For this reason, players were also instructed to land with extended legs and feet in order to not overestimate CMJ height. The mean values of force and velocity components of the individual loads required to calculate the F-V profiles were obtained from previously validated equations [2].

After jumping evaluations, participants performed 2 maximal 20-m sprints, with 5 min of rest between attempts, on a synthetic outdoor track. Both attempts were recorded by using an iPhone 7 (Apple Inc., Cupertino, CA, USA) and MySprint app (T-Mobile Inc., Bellevue, WA, USA). Athletes started from a crouching position (staggered-stance) with the right hand on the track. The start of the sprint was determined as the moment in which the right thumb of the athlete took off the ground (this was detected by visual inspection with MySprint). Five markers were located at 5, 10, 15, and 20 m to ensure that the split times were correctly recorded. Two independent observers were asked to select the first frame in which participants’ right thumb left the ground (i.e., start of the sprint) and, subsequently, the frame in which the pelvis was aligned with each of the four different markers for each of the recorded sprints [35]. Split times along with participants’ body mass and stature were used by the MySprint app to calculate F_0_, V_0_, P*_max_*, FV slope, RF peak, and DRF following previously validated formulas [2,35]. As sprinting and jumping F_0_, P*_max_*, and FV slope are calculated in relation to body mass and in subsequent analyses with these variables body mass bias was going to be statistically controlled for, these values were multiplied by body mass.

### 2.4. Statistical Analyses

Statistical analyses were performed using SPSS 24.0 (IBM, Armonk, NY, USA). Data were tested for normality of distribution and homogeneity of variances using a Kolmorov–Smirnov normality test and a Levene test, respectively. Linear regression assumptions were checked using residual vs. fitted, normal QQ, and Cook’s distance plots. There was no evidence of heteroscedasticity or multicollinearity. Given the association between body size and performance in adolescent males and because body mass and stature are highly related, residuals (individual values minus the mean) of the latter variables were obtained. Their product (i.e., residuals of body mass x residuals of stature) was calculated representing body size and it was used in subsequent partial correlation and multiple regression analyses so that collinearity among the independent variables was reduced [32]. Correlations were calculated between participants’ maturity offset and chronological age and both their jumping and sprinting performances, along with the components of their horizontal and vertical F-V profiles. They were analyzed twice. The first analysis corresponded to Pearson’s correlation analysis and the second one was a partial correlation analysis controlling for the effect of training experience (years of specific soccer training) and body size. Correlation effects were interpreted as small (0.10–0.29), moderate (0.30–0.49), large (0.50–0.69), and very large (≥0.70) [36]. Stepwise linear regression analyses were conducted between either participants’ maturity offset or chronological age (dependent variable) and parameters of the horizontal and vertical F-V profiles (independent variables). The same regression analyses were conducted including also body size and training experience as independent variables. Coefficients of determination (R^2^), unstandardized beta (regression) coefficients (B), *SE* of B (B *SE*), and standardized beta (regression) coefficients (β) were also calculated. Significance for all analyses was set at *p* < 0.05.

## 3. Results

Table 1 displays the descriptive data for chronological age, body mass, stature, sitting height, 5 m sprint time, 20 m sprint time, and CMJ height of participants for each category.

**Figure 1:**
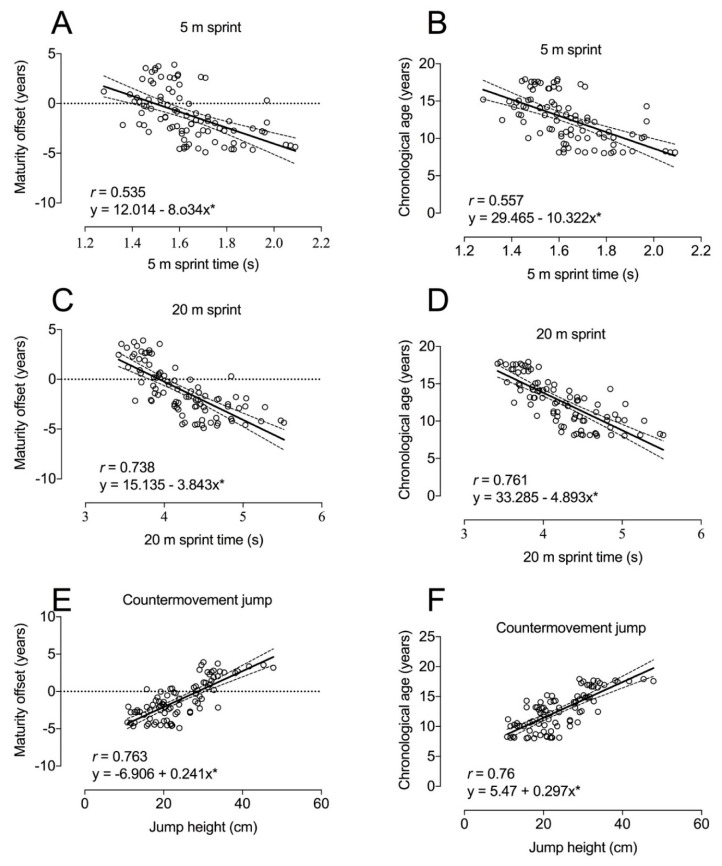
Associations between the performance in 5 m sprint, 20 m sprint, and countermovement jump, and maturity offset (**A**, **C** and **E**, respectively), and chronological age (**B**, **D** and **F**, respectively). Significant correlations: * *p* < 0.001.

Associations among performance variables and maturity offset and chronological age are shown in Figure 1. Very large correlations were identified between CMJ height and 20 m sprint times (Figure 1).

Partial correlations between 5 m, 20 m, and CMJ height and maturity offset controlling for training experience and body size were *r* = 0.23 *p* = 0.034), and 0.4 and 0.54 (*p* < 0.001), respectively. Partial correlations between 5 m, 20 m, and CMJ height and chronological age controlling for training experience and body size were *r* = 0.25 (*p* = 0.02), 0.45, and 0.56 *p* < 0.001), respectively.

Descriptive statistics of the different components of the vertical and horizontal F-V profiles, and Pearson correlation coefficients between them and both maturity offset and chronological are displayed in Table 2.

Both sprinting and jumping F-V parameters were similarly correlated with maturity offset and chronological age (Table 2). The largest correlations between parameters of the F-V profiles and both maturity offset and chronological age were identified in the F_0_ and P*_max_* of both horizontal and vertical F-V profile components regardless of the influence of both body size and experience. The F-V slope and V_0_ in sprinting exhibited very large and large correlations with maturity offset and chronological age with and without the influence of body size and experience, respectively. The other parameters showed lower correlation coefficients with maturity offset and chronological age (see Table 2).

Four stepwise regression analyses were conducted between participants’ maturity offset and chronological age, and the sprinting FV profile components (i.e., F_0_, V_0_, P*_max_*, FV slope, DRF, and RF peak), and the jumping FV profile components (i.e., F_0_, V_0_, P*_max_*, FV slope, and FV deficit) either with or without the inclusion of training experience and body size as predictors (see Table 3 and Table 4).

Sprinting F-V profile parameters combining P*_max_*, DRF, and F_0_, P*_max_*, and DRF were able to explain up to 94% and 91% of the total variability of maturity offset and chronological age, respectively. However, when including body size and training experience as possible predictors, P*_max_* and training experience explained 95% of the variability of both maturity offset and chronological age (see Table 3 and Table 4). Jumping F-V parameters combining F_0_ and FV slope were able to explain up to 95% of the total variability of both maturity offset and chronological age. However, when including body size and training experience as independent variables in the regression analysis, F_0_, training experience and FV slope were also able to explain up to 95% of the total variability of both maturity offset and chronological age. Sprinting and jumping F-V parameters showed similar relationships with both maturity offset and chronological age in the stepwise linear regression analyses (Table 3 and Table 4).

## 4. Discussion

The aims of this study were to determine the relationships between performance and the different components of the F-V profile in jumping and sprinting, and the maturity offset and chronological age of young soccer players, and to compare relationships between maturity offset and chronological age for jumping and sprinting. The main findings of the current research are that the horizontal F-V components F_0_, V_0_, P*_max_*, FV slope, and vertical F-V components F_0_ and P*_max_* displayed very large correlations with both chronological age and maturity offset regardless of the influence of body size and training experience. Additionally, different combinations of sprinting P*_max_*, DRF, F_0_, and training experience and body size explained more than 91% of the variations in maturity offset and chronological age. Furthermore, different combinations of jumping F_0_ and FV slope and training experience explained 95% of the variances in maturity offset and chronological age. In addition, and contrary to our hypothesis, similar correlations were found between sprinting and jumping performances, and components of the F-V profiles with either maturity offset or chronological age. 

Previously, Buchheit et al. [10] found that F_0_, V_0_, and P*_max_* were highly related to performance in acceleration and sprinting speed in players from U13 to U19 categories. The current results also confirm the relevance of these components, along with FV slope, on the development of sprinting performance in young soccer players as they showed very large correlations with maturity status and chronological age. According to the current results, F_0_, V_0_, and P*_max_* develop throughout the maturation process as long as sprinting performance, stature, weight, and muscle mass are increased due to the growing process [37]. Furthermore, the FV slope is decreased with maturation and age, thereby strengthening the relevance of F_0_ in contrast to V_0_ [2]. Therefore, the results of this study show a constant and homogeneous development in sprinting mechanical determinants such as P*_max_*, F_0_, V_0_, RF peak, and FV slope, but not in DRF. These results mean that older and more mature soccer players display better performance in these sprinting F-V components. Therefore, these F-V components can be considered very important and complementary markers of sprinting performance development, in order to improve the quality of training monitoring processes, rather than only evaluating sprinting times. Moreover, the combination of P*_max_*, DRF, and F_0_ was able to explain more than 91% of the variance in biological and chronological age, whereby P*_max_* was its highest predictor (Table 3). In addition, the inclusion of independent variables such as body size and training experience in the regression analysis improved its ability to predict both maturity offset and chronological age (Table 4), thereby also showing the influence of these variables on the sport development of young soccer players and more specifically on the development of sprinting F-V components [38,39]. 

Sprinting performance also displayed a very large correlation with maturity offset (Figure 1), confirming the findings by Méndez-Villanueva et al. [20], who found that sprinting ability in U14, U16, and U18 soccer players was highly related to the changes associated to biological maturation. Similar results were found in Australian footballers from U11 to U19 squads [40]. In contrast, McCunn et al. [41] found trivial and small correlation between maturation and sprinting speed (15 m) in U11, U12, and U13 soccer players. These different results may be due to the different statistical analyses conducted between these studies. Whereas McCunn et al. [41] found a lack of correlation between the maturity offset and sprint performance only in the U11–13 categories, in Gastin et al. [40] the correlations were conducted for the whole sample (U11−19 categories). Similarly, CMJ performance in the current study displayed a very large correlation with biological maturation (Figure 1), which is in agreement with a previous study by Lloyd et al. (2015) [23]. In any case, these results should be taken cautiously since these important correlations were substantially reduced when body size and training experience were included as covariables in the analysis. In addition, Murtagh et al. [29] found that 10 m and 20 m sprint performances differed between young soccer players and non-trained controls of similar maturation status, while CMJ height differed between groups only in the post- and mid-PHV subgroups. Accordingly, these authors suggested that vertical jumping ability should not be considered as a performance determinant in pre-PHV soccer players. In contrast, we found a large correlation between CMJ performance and maturity offset. In addition, jumping F_0_ and P*_max_* displayed very large correlation effects with maturity offset. These results mean that more mature and older soccer players display greater performance in these F-V components. This may be explained by the considerable increases in muscle mass observed at different ages [42], thereby generating a higher rate of development of these jumping determinants during the fastest stages of growth [43]. In addition, jumping F_0_ highly contributed to explain variability of both maturity offset and chronological age along with FV slope. Therefore, this result along with that similarly found in the correlation analysis indicate that F_0_ is the mechanical determinant of jumping performance that evolves to the greatest extent across the development process in young soccer players.

One of the novelties of the current study is the evaluation of the relationships between F-V parameters in both jumping and sprinting and chronological age. The fact that similar relationships were found between these variables and maturity offset and chronological age is in contrast to previous research which highlighted the need to relate performance development to maturation status in order to determine talent identification markers [17,41]. Whereas growth and strength development are not entirely associated with chronological age evolution, especially at some specific stages [17,41], according to the present results, it seems that these differences are not enough to be taken into consideration throughout the whole development process. Accordingly, Mendez-Villanueva et al. [20] found significant differences between young soccer players of different categories (i.e., chronological ages) in sprint performance, but when controlling for maturation status, these differences disappeared. Thus, biological and chronological ages similarly influence the evolution of sprinting capabilities in young soccer players.

A limitation of the current study is that only players from the same academy were evaluated, therefore these results should be extrapolated to other contexts with caution.

## 5. Practical Applications and Conclusions

The frequent application of jumping and linear sprinting tests to obtain the force-velocity profiles may provide useful information in young soccer players. In this sense identification of some sprint and jumping mechanical determinants such as F_0_, V_0_, P*_max_* and FV slope, and F_0_ and P*_max_*, respectively, at a specific age or maturation status in young soccer players, may assist to strengthen those components of the F-V profile which are weaker throughout the training process. Accordingly, some training methods could be implemented in the training regime of young soccer players, such as resisted sprint training (with sled or vest) or assisted sprint training (with the use of bungee cords or an assistance pulley), which promote the development of the horizontal strength component across the entire spectrum [44,45,46] and provide an orientation towards either strength or speed depending on the specific needs of the athlete [47]. However, some jumping F-V mechanical determinants such as V_0_ are not expected to increase with the maturation process or age. In addition, the results of the present study suggest that sprinting and jumping capabilities in young soccer player can be indistinctly monitored with respect to their chronological age or to maturity offset. As an example, a 15 year-old soccer player who did not improve V_0_ after the pre-season, despite improving the sprinting time, should focus on developing this component through the implementation of specific training exercises such as those previously mentioned (e.g., unloaded maximal sprints) thus correcting the F-V imbalance, independently of the maturational status. Combining sprinting P*_max_*, DRF, F_0_, jumping F_0_, and FV slope, in addition to body size and training experience, can explain, to a very great extent, the changes in both maturity offset and chronological age. Sprint and CMJ performances and sprinting and jumping components of the F-V profiles correlate similarly with both maturity offset and chronological age in young soccer players. Further studies may focus on the analysis of the differences of sprinting and jumping F-V profile components between pre-, mid-, and post-pubertal young soccer players

## Figures and Tables

**Table 1:** Descriptive statistics (mean ± standard deviation) of chronological age, body mass, stature, sitting height, 5 m sprint time, 20 m sprint time, and countermovement jump (CMJ) height of participants for each category.

Category	*n*	Age (years)	Weight (kg)	Stature (cm)	Sitting Height (cm)	5 m Time (s)	20 m Time (s)	CMJ Height (cm)
U 18	18	17.27 ± 0.46	71.78 ± 11.37	177.39 ± 6.89	91.72 ± 3.04	1.55 ± 0.07	3.70 ± 0.14	35.0 ± 5.34
U 16	17	14.54 ± 0.45	61.12 ± 12.63	164.53 ± 5.0	87.98 ± 2.67	1.51 ± 0.15	3.98 ± 0.30	25.92 ± 4.97
U 14	17	12.71 ± 0.42	44.48 ± 7.06	151.47 ± 6.03	79.72 ± 3.17	1.61 ± 0.15	4.23 ± 0.36	22.35 ± 5.02
U 12	19	10.55 ± 0.44	43.58 ± 8.58	144.42 ± 6.19	75.22 ± 3.23	1.70 ± 0.14	4.52 ± 0.37	18.45 ± 5.83
U 10	18	8.40 ± 0.41	29.72 ± 5.17	129.0 ± 4.49	66.15 ± 2.30	1.78 ± 0.16	4.70 ± 0.40	18.06 ± 4.84

U number: under specific chronological age.

**Table 2:** Descriptive statistics of parameters of the force-velocity profiles in sprinting and vertical jumping, and correlation coefficients and *p*-values between these parameters, either not controlling for or considering the influence of training experience and body size, and both maturity offset and chronological age (CA).

			Maturity Offset				CA			
			Bivariate r		Partial r		Bivariate r		Partial r	
**Sprinting**	**Mean**	**SD**	*r*	*p*	*r*	*p*	*r*	*p*	*r*	*p*
F_0_ (N)	309.43	132.15	0.89	<0.001	0.7	<0.001	0.85	<0.001	0.62	<0.001
V_0_ (m·s^−1^)	6.57	1.06	0.77	<0.001	0.45	<0.001	0.79	<0.001	0.51	<0.001
P_max_ (W)	530.41	286.63	0.92	<0.001	0.78	<0.001	0.9	<0.001	0.73	<0.001
FV slope (N·s·m^−1^)	2572.87	1714.82	−0.79	<0.001	−0.56	<0.001	−0.74	<0.001	−0.51	<0.001
DRF (%)	–0.09	0.02	0.29	0.005	0.18	0.098	0.29	0.007	0.2	0.065
RF peak (%)	0.43	0.05	0.59	<0.001	0.26	0.015	0.61	<0.001	0.3	0.005
**Jumping**	**Mean**	**SD**	*r*	*p*	*r*	*p*	*r*	*p*	*r*	*p*
F_0_ (N)	1407.2	618.21	0.91	<0.001	0.79	<0.001	0.89	<0.001	0.75	<0.001
V_0_ (m·s^−1^)	3.62	1.08	0.23	0.034	0.11	<0.001	0.21	0.226	0.07	0.508
P_max_ (W)	1277.69	695.75	0.87	<0.001	0.67	<0.001	0.83	<0.001	0.6	<0.001
FV slope (N·s·m^−1^)	2292.78	1300.53	−0.01	0.916	−0.03	0.771	−0.0	0.519	−0.01	0.923
FV deficit (%)	8.63	3.69	0.49	<0.001	0.17	0.122	−0.09	0.39	0.11	0.328

SD, standard deviation; r, Pearson’s correlation coefficient; F_0_, theoretical maximal force; V_0_, theoretical maximal velocity; FV slope, force-velocity slope; P*_max_*, theoretical maximal power; FV deficit, percent difference respect to the optimal FV profile; DRF, decrease in the ratio of horizontal force; RF peak: maximal ratio of horizontal force; Bivariate *r*: bivariate Pearson’s correlation; Partial *r*: partial correlation controlling for training experience (years of specific soccer training) and body size (product of body mass and stature residuals [individual values minus the mean]);. Statistical significance set at *p* < 0.05.

**Table 3:** Stepwise regression analyses relating both maturity offset and chronological age (CA) with components of force-velocity (FV) profiles in sprinting and jumping.

Sprinting	Model 1			Model 2			Model 3		
**Maturity offset**	B	SE B	β	B	SE B	β	B	SE B	β
P*_max_*	0.01	0	0.92 ^§^	0.01	0	0.9 ^§^	0.003	0.002	0.35 ^§^
DRF				22.65	6.36	0.14 ^†^	38.94	8.5	0.24 ^§^
F_0_							0.11	0.004	0.55 ^†^
*R*^2^ adjusted	0.849			0.867			0.877		
**CA**	B	SE B	β	B	SE B	β	B	SE B	β
P*_max_*	0.01	0	0.9 ^§^	0.01	0	0.87 ^§^			
DRF				27.57	9.24	0.14 ^†^			
*R*^2^ adjusted	0.8			0.817					
**Jumping**	**Model 1**			**Model 2**					
Maturity offset	B	SE B	β	B	SE B	β			
F_0_	0.004	0	0.91 ^§^	0.004	0	0.96 ^§^			
FV slope				−0.15	0.03	−0.23 ^§^			
*R*^2^ adjusted	0.834			0.882					
**CA**	B	SE B	β	B	SE B	β			
F_0_	0.004	0	0.89 ^§^	0.005	0	0.94 ^§^			
FV slope				−0.18	0.04	−0.21 ^§^			
*R*^2^ adjusted	0.792			0.831					

FV slope, force-velocity slope; V_0_, theoretical maximal velocity; F_0_, theoretical maximal force; P*_max_*, theoretical maximal power; FV deficit, percent difference respect to the optimal FV profile; R^2^ = Pearson’s multivariate coefficient of determination; B = unstandardized beta (regression) coefficient; SE B = standard error of B; β = standardized beta (regression) coefficient. ^†^ *p* < 0.01, ^§^ *p* < 0.001.

**Table 4:** Stepwise regression analyses relating both maturity offset and chronological age (CA) with components of force-velocity (FV) profiles in sprinting and jumping, body size, and training experience

Sprinting	Model 1			Model 2			Model 3		
**Maturity offset**	B	SE B	β	B	SE B	β	B	SE B	β
P*_max_*	0.01	0	0.92 ^§^	0.01	0	0.64 ^§^	0.01	0.001	0.76 ^§^
Training experience				0.39	0.06	0.36 ^§^	0.38	0.06	0.36 ^§^
RF peak							−7.76	2.22	−0.16 ^†^
*R*^2^ adjusted	0.849			0.897			0.909		
**CA**	B	SE B	β	B	SE B	β	B	SE B	β
P*_max_*	0.01	0	0.9 ^§^	0.01	0	0.55 ^§^	0.01	0	0.62 ^§^
Training experience				0.58	0.08	0.43 ^§^	0.53	0.08	0.4 ^§^
Body size							0.12	0.04	0.12 *
*R*^2^ adjusted	0.8			0.87			0.881		
**Jumping**	**Model 1**			**Model 2**					
Maturity offset	B	SE B	β	B	SE B	β	B	SE B	β
F_0_	0.004	0	0.91 ^§^	0.003	0	0.7 ^§^	0.003	0	0.74 ^§^
Training experience				0.43	0.06	0.4 ^§^	0.36	0.05	0.33 ^§^
FV slope							−0.11	0.2	−0.17 ^§^
*R*^2^ adjusted	0.834			0.901			926		
**CA**	B	SE B	β	B	SE B	β	B	SE B	β
F_0_	0.004	0	0.89 ^§^	0.003	0	0.54 ^§^	0.003	0	0.62 ^§^
Training experience				0.61	0.08	0.46 ^§^	0.54	0.07	0.4 ^§^
FV slope							−0.12	0.3	−0.14 ^§^
*R*^2^ adjusted	0.792			0.880			0.896		

FV slope, force-velocity slope; V_0_, theoretical maximal velocity; F_0_, theoretical maximal force; P*_max_*, theoretical maximal power; FV deficit, percent difference respect to the optimal FV profile; Training experience: years of specific soccer training; Body size: product of body mass and stature residuals (individual values minus the mean); R^2^ = Pearson’s multivariate coefficient of determination; B = unstandardized beta (regression) coefficient; SE B = standard error of B; β = standardized beta (regression) coefficient. * *p* < 0.05, ^†^ *p* < 0.01, ^§^ *p* < 0.001.
